# The effects of Nordic walking on cognitive function in older adults: a systematic review and meta-analysis

**DOI:** 10.3389/fnagi.2025.1666449

**Published:** 2025-09-17

**Authors:** Haobai Li, Ke Zhu, Jianyu Gan, Ziyi Wang, Zhikun Gao, Liangru Liu, Xiaojie Guo, Jianfeng Niu

**Affiliations:** ^1^Sports Coaching College, Beijing Sport University, Beijing, China; ^2^School of Physical Education, Guangxi University, Guangxi, China; ^3^Sports Department, Nankai University, Tianjin, China

**Keywords:** Nordic walking, exercise, cognitive function, older adults, meta-analysis

## Abstract

**Objectives:**

Nordic walking (NW), as a specialized form of aerobic exercise, emerges as a promising strategy to improve the cognitive function in older population. However, the effectiveness of NW has yet to be definitively confirmed due to the variances in the study designs and observations. This systematic review and meta-analysis was thus conducted to examine the effect of NW interventions on cognitive function of older adults.

**Methods:**

The search was conducted in August 2025 on Web of Science, PubMed, SPORT-Discus, Medline, the Cochrane Library, Scopus, and PsycINFO databases. Two reviewers independently reviewed the search results, extracted the data, and assessed the risk of bias and certainty of evidence. Meta-analyses and meta-regressions were performed to determine the overall effect size and the impact of potential moderators.

**Results:**

Initial screening identified 336 records, and after full-text assessment, eight studies (from 2014 to 2024) comprising 327 participants (71.19 ± 5.44 yrs) were included. The effect size of NW on executive function was significant [Hedges’ *g* = 0.89, 95% CI (0.27, 1.50), *p* = 0.01], while the effects were non-significant for global function, memory function, attention, information processing, and perceptual ability (*p* > 0.05). Subgroup analysis indicated that the health conditions of participants and the types of control groups significantly moderated executive function. Specifically, NW showed significant improvements (i) in older adults with health conditions and (ii) compared with inactive control groups (*p* = 0.04). Meta-regression revealed a significant positive correlation between the total intervention time of NW and its effect size (*p* < 0.01).

**Conclusion:**

This systematic review and meta-analysis demonstrates that NW interventions could improve executive function in older adults, especially those with health conditions.

**Systematic review registration:**

https://www.crd.york.ac.uk/prospero, identifier CRD42025638467.

## Introduction

Aging is associated with progressive physiological changes, including reduced neurogenesis, impaired synaptic plasticity, and decreased cerebral perfusion in older adults (Castellano et al., 2017; Poulose et al., 2017; Raichlen and Alexander, 2017; Gonzales et al., 2022). These changes collectively contribute to cognitive decline, particularly affecting memory, executive function, and processing speed (Bettio et al., 2017; Fan et al., 2017; Leeuwis et al., 2017; Huang et al., 2022). The resultant cognitive decline in the older population leads to profound adverse consequences, including a marked deterioration in quality of life, increased dependency on caregiving support, and a higher risk of neurodegenerative diseases, particularly Alzheimer’s disease (Li et al., 2017; Sáez De Asteasu et al., 2017). Given these impacts, developing effective interventions to maintain or improve cognitive function in older adults remains essential.

Current approaches against cognitive decline primarily encompass pharmacological interventions, cognitive training paradigms, and lifestyle modifications (Srikanth et al., 2020; Barnes et al., 2023; Antonenko et al., 2024; Faraziani and Eken, 2024). While these strategies demonstrate some efficacy, they are constrained by some limitations. Specifically, pharmacological interventions are frequently associated with adverse side effects and potential long-term complications, raising concerns about their safety profile and sustainability (Parnetti et al., 1997; Blackman et al., 2021; Van Dyck et al., 2023). Cognitive training approaches, though theoretically promising, often exhibit limited ecological validity and practical applicability, with questionable generalizability to real-world cognitive functions (Bahar-Fuchs et al., 2013; Butler et al., 2018). Lifestyle modifications face challenges in implementation and long-term adherence, particularly in elderly populations with varying health conditions and functional capacities (Knight et al., 2016; Barber et al., 2023). These limitations highlight the need for developing alternative intervention strategies that are not only efficacious but also characterized by enhanced safety, accessibility, and sustainability.

Recent literature increasingly corroborates that exercise is a particularly promising intervention strategy (Sanders et al., 2020; Zhang et al., 2023; Hatami et al., 2025). Exercise has been shown to promote neuroplasticity and regulate inflammatory processes, thereby creating an optimal neurobiological environment for cognitive preservation and enhancement in older populations (Hortobágyi et al., 2022; Vints et al., 2024; Lavie et al., n.d.). Among various forms of exercise, walking is the preferred choice for most older adults to enhance their cognitive function due to its safety and low intensity, especially for those with limited physical function associated with aging or disease (Adderley et al., 2025; Ahmadpour et al., 2025; Cunha et al., 2025; Sandroff et al., 2025). Notably, recent research has shown that compared with standard walking, a form of walking known as Nordic walking (NW) is less physically demanding for older adults and may provide greater cognitive benefits (Passos-Monteiro et al., 2020; Nemoto et al., 2021; Kettinen et al., 2023). NW distinguishes itself through the incorporation of two specially designed poles that facilitate active engagement of the upper body musculature, thereby resulting in more propulsion and energy expenditure (Schiffer et al., 2006). The arm-swinging motion involved in NW is beneficial for maintaining coordination of upper and lower limbs, which may generate similar cognitive effects to dual-task walking (Doi et al., 2014; Franzoni et al., 2018; Gomeñuka et al., 2019). Studies have demonstrated that NW induced significant improvements in executive function and memory of older adults with or without health conditions as compared to walking or blank control groups (Passos-Monteiro et al., 2020; Guszkowska et al., 2022; Kettinen et al., 2023; Ploydang et al., 2023). However, despite these promising preliminary results, existing evidence remains inconsistent. Several studies reported that no significant changes in cognitive function were observed following NW intervention (Passos-Monteiro et al., 2020; Haas et al., 2024). These inconsistencies may stem from variations in study design, such as intervention duration, intensity, or participant characteristics. Additionally, the lack of standardized protocols for NW and the heterogeneity in cognitive function assessment metrics further complicate the interpretation of study findings.

Therefore, to highlight the recent study findings and explicitly and comprehensively examine the effects of NW on cognitive function in older adults and the potential contributors to such effects, we completed a systematic review and meta-analysis based upon up-to-date peer-reviewed publications. This work will ultimately provide critical knowledge to inform the appropriate intervention design in future research and rehabilitative practice for the maintenance of cognitive function in older populations.

## Materials and methods

### Study protocol

This systematic review and meta-analysis was conducted using Preferred Reporting Items for Systematic Reviews and Meta-Analysis (PRISMA) guidelines (Page et al., 2021) and registered with PROSPERO (Registration ID: CRD42025638467), an international prospective registry for systematic reviews.

### Literature search

Two authors (HL and JG) independently searched Web of Science, PubMed, MEDLINE, SPORT-Discus, Cochrane Library, Scopus, and PsycINFO from inception to August 5, 2025; Studies were searched in the electronic databases using the following key terms combined by Boolean logic (“AND”, “OR”): (“Nordic walking” OR “Nordic pole walking” OR “pole walking”) AND (“Cognitive function” OR “cognition” OR “Cognitive performance”). A secondary search strategy was also used, which involved a manual search in the reference lists of eligible studies (i.e., citation tracking). Searches were limited to publications in English. Any disagreements arising during this process were resolved through discussion between the two authors (HL and JG), with additional input provided by a third author (JN). Detailed search strategies are provided in Supplementary Table 1.

### Selection criteria

All included studies must be published articles. The inclusion criteria were carried out according to the PICOS principle: (1) Population: All the participants included in this study were at least 60 years old. None of them had used any drugs known to significantly affect cognitive function, or had discontinued such drugs for more than 4 weeks, or had indicated their actual medication status at baseline to ensure the comparability of drug exposure between the two groups; (2) Interventions: the interventions used were NW only or NW combined with other interventions. When NW was used in combination with other interventions, the control group was supposed to receive other interventions alone to ensure that the observed changes were caused by NW; (3) Comparisons: Each group is characterized as either active (e.g., interventions involving physical activities other than Nordic walking, NW) or inactive (e.g., non-intervention, or routine treatment for the diseases the subjects suffer from); (4) Outcomes: Outcome measures reflecting cognitive function were employed. (5) Study design: The study employed randomized controlled trials or randomized crossover trial designs. Articles with the following conditions will be excluded: (1) did not investigate cognitive function outcomes or provide specific data of outcome measures (e.g., reporting only *p*-values without means/SDs); (2) review papers, conference abstracts, and articles; (3) those with duplicate publications; (4) non-English publications.

### Data extraction

The process of data extraction was conducted independently by two authors (HL and JG) according to the Cochrane Collaboration Handbook. The extracted information of the publications included: study (authors, year), participants (age, sex, physical condition), grouping and sample size, interventions (type, frequency, number of sessions, duration of each session, duration of intervention), auxiliary means (e.g., Nordic walking poles or Hiking pole, etc.), and outcome measures. Any outcome measures on which the two authors disagreed were discussed with the other two authors (JN and KZ) until a consensus was achieved. For each study, extract the mean and standard deviation (SD) of the post-intervention indicator results. For studies that do not report changes in results before and after, or those presenting results in the form of “Mean ± SE/SEM (Standard Error/Standard Error of the Mean)”, use the following formula for calculation (Borenstein et al., 2013):


$$\mathrm{SD}\,\left(\mathrm{pre}\right)=\mathrm{SE}\,\left(\mathrm{pre}\right)\,\sqrt{n}=\times ;\mathrm{SD}\,\left(\mathrm{post}\right)=\mathrm{SE}\,\left(\mathrm{post}\right)\,\sqrt{n}$$

R=0.4/0.5

When the full-text article data were presented only in a figure format, WebPlotDigitizer (Ankit Rohatgi, 2019, V.4.2; WebPlotDigitizer, Pacifica, CA, USA) was used to extract the data from the figures. In the absence of any relevant data, the first author or the corresponding author of the article would be contacted via email to obtain the required data.

### Quality assessment

The quality of the included studies was independently evaluated by two authors (HL and JG) according to the principles of the Physiotherapy Evidence Database (PEDro). The PEDro scale, specifically designed to assess the methodological quality of randomized controlled trials in physical interventions, is highly suitable for evaluating the studies in this research (de Morton, 2009; Cashin and McAuley, 2020). This scale examines crucial elements such as randomization, blinding, and allocation concealment, which are essential for ensuring the internal validity of the studies included in this systematic review and meta-analysis (Cashin and McAuley, 2020).

Specifically, the PEDro scale consists of 11 items, to which we were required to respond with “no” or “yes”. For each “no” response, a value of 0 was assigned, and for each “yes” response, a value of 1 was assigned. The total score for each study ranged from 0 to 11. Since blinding (especially of participants and investigators) is difficult to implement in exercise intervention trials (Sherrington et al., 2010), the methodological quality classification of each article was adjusted, taking into account the eligibility criteria as previously described [sum scores: ≥6 (“high quality, low risk of bias”); scores: 4–5 (“acceptable quality, moderate risk of bias”); scores: ≤ 3 (“low quality, high risk of bias”)].

The quality of the evidence was also independently appraised by two authors (HL and JG) based on the Grading of Recommendations Assessment, Development and Evaluation (GRADE) criteria. The GRADE criteria characterize the evidence in terms of study limitations, imprecision, inconsistency, indirectness, and publication bias. In cases where the two authors disagreed on any score, a third author (JN or KZ) was consulted for discussion until a consensus was reached.

### Statistical analysis

Meta-analysis was carried out using Stata/MP 17.0 (STATA Corp, College Station, TX, USA) and R 4.2.0 (R Core Team, R Foundation for Statistical Computing, Vienna, Austria). Given the diverse measurement units of outcome measures across studies, such as time, score, number of stimuli, etc., a random-effects model was employed to calculate Hedges’ g and the 95% confidence interval (CI), which served as the indicator of the effect size for the difference in pre-post changes between the intervention and control groups (Fritz et al., 2012). The effect sizes were categorized as follows: trivial (Hedges’ *g* < 0.2), small (0.2 ≤ Hedges’ *g* < 0.5), moderate (0.5 ≤ Hedges’ *g* < 0.8), or large (Hedges’ *g* ≥ 0.8) (Cohen, 2013). Additionally, prediction intervals were computed to reflect heterogeneity in comparison with confidence intervals. The between-study variance was estimated using the restricted maximum likelihood estimator with Hartung-Knapp adjustment (Veroniki et al., 2016). Statistical heterogeneity was evaluated by means of the heterogeneity chi-squared (χ^2^) and I^2^ values. The degree of heterogeneity was interpreted in accordance with the guidelines of the Cochrane Collaboration: 0%–40% might not be of significance; 30%–60% may indicate moderate heterogeneity; 50%–90% may represent substantial heterogeneity; and 75%–100% implies considerable heterogeneity (Higgins, 2003). In the event of substantial or considerable heterogeneity (I^2^ > 50%), subgroup analyses were conducted to explore the impact of study characteristics (e.g., the physical condition of the participants). Additionally, we performed meta-regression to explore the dose-response relationship of NW on cognitive function. The intervention duration, session number, and total time (i.e., session number × session duration) were used as the effect moderators. Subsequently, sensitivity analyses were performed to assess the stability of the pooled estimates and to determine whether any study influenced the overall effect size (Veroniki et al., 2016). Moreover, publication bias was assessed through the generation of funnel plots and the conduct of Egger’s test. If significant asymmetry was detected, the Trim and Fill method was utilized to adjust for publication bias (Duval and Tweedie, 2000).

## Results

### Study selection

**FIGURE 1:**
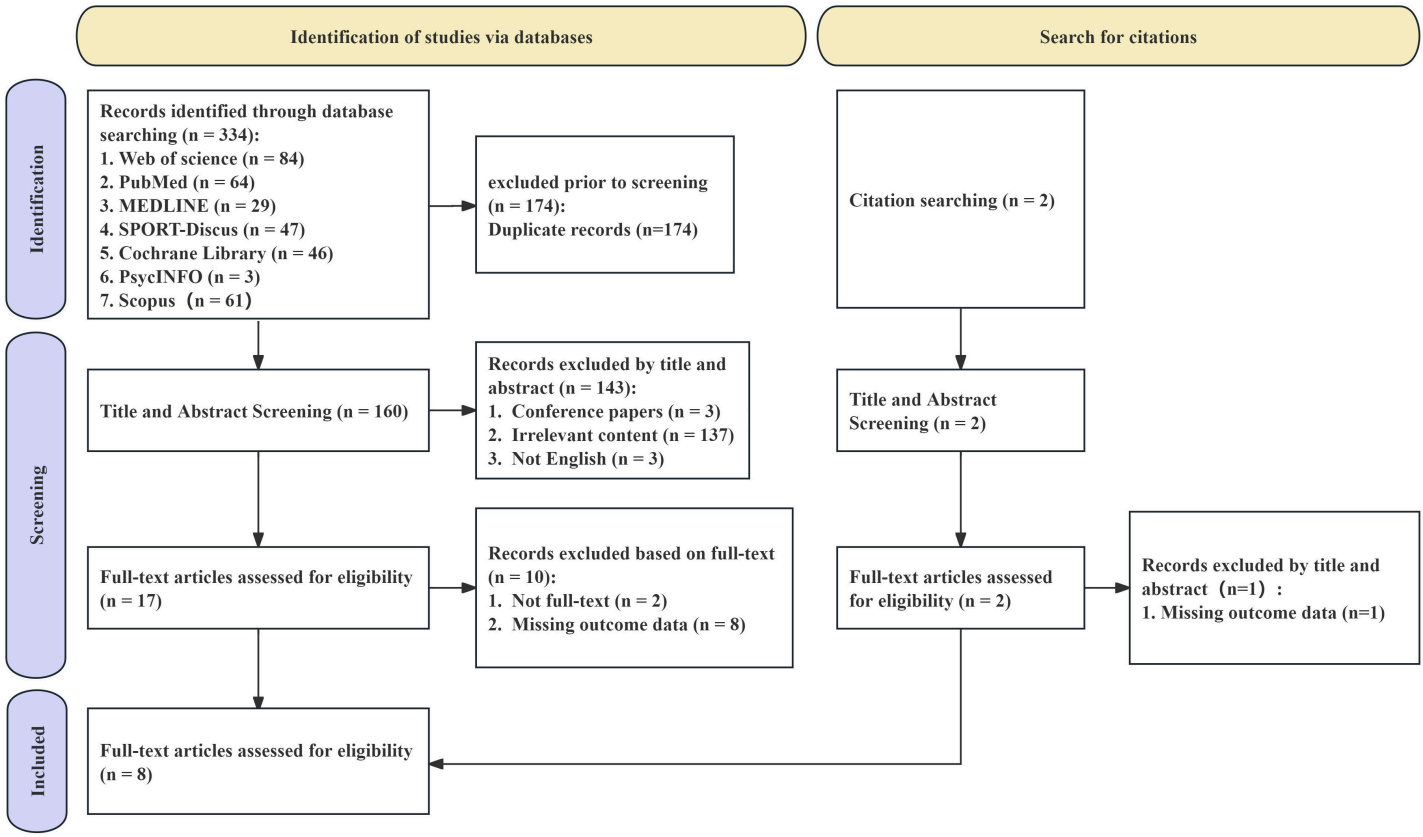
Flow diagram of the study selection process.

The results of the study selection process are summarized in a PRISMA flowchart (Figure 1). The study selection commenced with the identification of 336 records through database searches, which were distributed among Web of Science (*n* = 84), PubMed (*n* = 64), MEDLINE (*n* = 29), SPORT-Discus (*n* = 47), Cochrane Library (*n* = 46), Scopus (*n* = 61), and PsycINFO (*n* = 3). Additionally, two further studies were identified through citation searching. After removing 174 duplicate records, a total of 162 records were available for the screening phase. During the title and abstract screening stage, 143 records were excluded. Subsequently, a full-text eligibility assessment was conducted on 18 articles, and 10 studies were excluded. Eventually, 8 studies were included in the meta-analysis.

### Characteristics of included studies

#### Participant characteristics

The eight studies included in the analysis were conducted across seven countries: Germany (*n* = 1), Poland (*n* = 1), Japan (*n* = 1), Thailand (*n* = 1), Brazil (*n* = 2), Italy (*n* = 1), and Finland (*n* = 1). The total number of participants across all studies was 327. Excluding one study that did not report age information (Ebersbach et al., 2014), the mean age of participants in the remaining seven studies was 70.92 years (Table 1). Six studies included both male and female participants (Passos-Monteiro et al., 2020; Miyazaki et al., 2022; Angiolillo et al., 2023; Kettinen et al., 2023; Ploydang et al., 2023; Haas et al., 2024), one study exclusively involved female participants (Guszkowska et al., 2022), and one study did not report the gender distribution of participants (Ebersbach et al., 2014).

**TABLE 1:** Characteristics of included studies (*n* = 8).

References	Sample size	Age	Sex men/women	Physical condition	Interventions	Duration	Frequency	Session number	Session duration	Outcome measures
Ebersbach et al., 2014	NW (19)	Unclear	Unclear	Parkinson’s disease	Nordic walking	8 weeks	2 times/week	16	60 min	Information processing speed: cRT↑; nRT→
	CG (19)				Domestic exercise					
Passos-Monteiro et al., 2020	NW (16)	64.9 ± 10.2	13/3	Parkinson’s disease	Nordic walking	9 weeks	2 times/week	18	The first 3 weeks: 35 min The last 6 weeks: 40–60 min	Global cognition: MoCA→
	CG (17)	70.5 ± 5.8	7/10		Free walking					
Guszkowska et al., 2022	NW (20)	80.25 ± 5.755	0/20	Essential health	Nordic walking	3 months	2 times/week	24	60 min	Information processing speed: APT (3/8) perception speed↑Perceptual Abilities: APT (3/8) perception fallibility↑Attention: APT (3/8) attention fallibility↑
	CG (20)		0/20		No intervention					
Miyazaki et al., 2022	NW (29)	67.93 ± 5.81	22/7	Essential health	Nordic walking combined with a daily supplement containing 8 g of protein	4 weeks	3 times/week	9∼12	45 min	Global cognition: MoCA→Executive functions: FAB↑
	CG (29)		23/6		Take a daily protein supplement containing about 8 grams of protein					
Angiolillo et al., 2023	NW (9)	78.89 ± 6.68	3/6	Alzheimer’s disease	Nordic walking combined with reality orientation therapy, music therapy, motor, proprioceptive and postural rehabilitation	24 weeks	2 times/week	48	60 min	Global cognition: MMSE→Executive functions: FAB→; SWCT-time↑memory: RVLT-I→; RVLT-D↑Perceptual Abilities: CGD→Attention: Attentional Matrices→Fluid Intelligence: CPM↑
	CG (13)	78.92 ± 8.04	5/8		Reality orientation therapy, music therapy, motor, proprioceptive and postural rehabilitation					
Kettinen et al., 2023	NW (25)	69 ± 4.4	16/9	Essential health	Nordic walking	——	——	1	——	Information processing speed: TMT-A↑Executive functions: TMT(B-A)↑; TMT-B↑
	CG (25)				Walking					
Ploydang et al., 2023	NW (17)	69.2 ± 5.3	5/11	Type 2 Diabetes	Aquatic Nordic walking	12 weeks	3 times/week	36	60 min	Global cognition: MoCA↑; MMSE→Executive functions: SCT→; TMT-B→
	CG (17)	68.9 ± 3.7	7/10		No intervention					
Haas et al., 2024	NW (31)	67.87 ± 11.2	23/8	Parkinson’s disease	Nordic walking	12 weeks	2 times/week	24	60 min	Global cognition: MoCA→
	CG (21)	66.76 ± 8.97	17/4		Deep-water exercise					

APT(3/8), Attention and Perceptivity Test, version 3/8; CGD, Copying Geometric Drawings; CPM, Raven’s Colored Progressive Matrices; cRT, Cued reaction time; FAB: Frontal Assessment Battery; MoCA: Montreal Cognitive Assessment; MMSE: Mini Mental State Examination; nRT: noncued reaction time; RVLT-I, Rey’s auditory Verbal Learning Test-Immediate Recall; RVLT-D, Rey’s auditory Verbal Learning Test-Delayed Recall; SCT, Stroop Color test; SWCT-time, Stroop Word-Color Interference test; TMT-A, Trail Making Test part A; TMT-B, Trail Making Test part B; TMT (B-A), Trail Making Test part B-A; ↑, Intervention significantly (*p* < 0.05) improved the outcome compared with control; →, Intervention induced no significant difference compared with control (*p* > 0.05).

Participants in three studies were essential health (Guszkowska et al., 2022; Miyazaki et al., 2022; Kettinen et al., 2023), while the other five studies included participants with health conditions (Ebersbach et al., 2014; Passos-Monteiro et al., 2020; Angiolillo et al., 2023; Ploydang et al., 2023; Haas et al., 2024), including Parkinson’s disease (*n* = 3) (Ebersbach et al., 2014; Passos-Monteiro et al., 2020; Haas et al., 2024), Type 2 diabetes (*n* = 1) (Ploydang et al., 2023), and Alzheimer’s disease (*n* = 1) (Angiolillo et al., 2023).

#### Intervention characteristics

Of the eight studies incorporated in this review, seven were conducted using a randomized controlled trial design (Passos-Monteiro et al., 2020; Nemoto et al., 2021; Guszkowska et al., 2022; Miyazaki et al., 2022; Angiolillo et al., 2023; Ploydang et al., 2023; Haas et al., 2024), with the remaining study adopting a randomized crossover controlled trial design (Kettinen et al., 2023). The characteristics of the intervention measures are detailed in Table 1. Among the included studies, five studies independently employed NW as the intervention measure (Ebersbach et al., 2014; Passos-Monteiro et al., 2020; Kettinen et al., 2023; Haas et al., 2024, 2024). One study combined NW with daily protein supplementation (Miyazaki et al., 2022), while another study utilized aquatic NW as the intervention (Ploydang et al., 2023). Additionally, one study implemented a multimodal intervention approach, integrating NW with reality orientation therapy, music therapy, physical exercise, proprioceptive training, and postural rehabilitation (Angiolillo et al., 2023).

Regarding the control groups, three studies employed non-active control conditions (Guszkowska et al., 2022; Miyazaki et al., 2022; Ploydang et al., 2023), which included maintaining usual daily activities (*n* = 2) (Guszkowska et al., 2022; Ploydang et al., 2023) and nutritional supplementation (daily intake of 8g protein supplements) (*n* = 1) (Miyazaki et al., 2022). The remaining five studies utilized active control conditions (Ebersbach et al., 2014; Passos-Monteiro et al., 2020; Angiolillo et al., 2023; Kettinen et al., 2023; Haas et al., 2024), which involved interventions such as domestic exercise (*n* = 1) (Ebersbach et al., 2014), deep-water exercise (*n* = 1) (Haas et al., 2024), regular walking (*n* = 2) (Passos-Monteiro et al., 2020; Miyazaki et al., 2022), and a multimodal training program incorporating reality orientation therapy, physical exercise, and proprioceptive training (*n* = 1) (Angiolillo et al., 2023).

The duration of a single intervention session varied from 35 to 60 min. The frequencies of intervention included a single session (*n* = 1) (Kettinen et al., 2023), two sessions per week (*n* = 5) (Ebersbach et al., 2014; Passos-Monteiro et al., 2020; Guszkowska et al., 2022; Angiolillo et al., 2023; Haas et al., 2024), and three sessions per week (*n* = 2) (Miyazaki et al., 2022; Ploydang et al., 2023).

The overall intervention period ranged from 1 day to 24 weeks, and the total number of interventions across the studies was between 1 and 36. Specifically, the intervention protocols varied across the studies: one studies involved 16 sessions over 8 weeks (Ebersbach et al., 2014), one study included 18 sessions over 9 weeks (Passos-Monteiro et al., 2020), one study implemented 24 sessions over 3 months (Guszkowska et al., 2022), one study featured 9–12 sessions over 4 weeks (Miyazaki et al., 2022), one study consisted of 48 sessions over 24 weeks (Angiolillo et al., 2023), one study implemented 24 sessions over 24 weeks (Haas et al., 2024), one study involved a single session conducted over 1 day (Kettinen et al., 2023), and one study provided 36 sessions over 12 weeks (Ploydang et al., 2023). Across all studies, participants received prior instruction on the knowledge and skills necessary for NW before commencing the interventions.

### Study outcomes

This meta-analysis included 23 effect sizes from 8 studies. These effect sizes encompassed multiple domains of cognitive ability, including global cognitive function (Passos-Monteiro et al., 2020; Miyazaki et al., 2022; Angiolillo et al., 2023; Ploydang et al., 2023; Haas et al., 2024), memory function (Angiolillo et al., 2023), executive function (Miyazaki et al., 2022; Angiolillo et al., 2023; Kettinen et al., 2023; Ploydang et al., 2023), perceptual abilities (Guszkowska et al., 2022; Angiolillo et al., 2023), information processing speed (Guszkowska et al., 2022; Kettinen et al., 2023), and attention (Ebersbach et al., 2014; Guszkowska et al., 2022; Angiolillo et al., 2023). Specific cognitive tests administered and their corresponding domain classifications are detailed in Supplementary Table 3.

### Effect of NW on cognitive function levels

**FIGURE 2:**
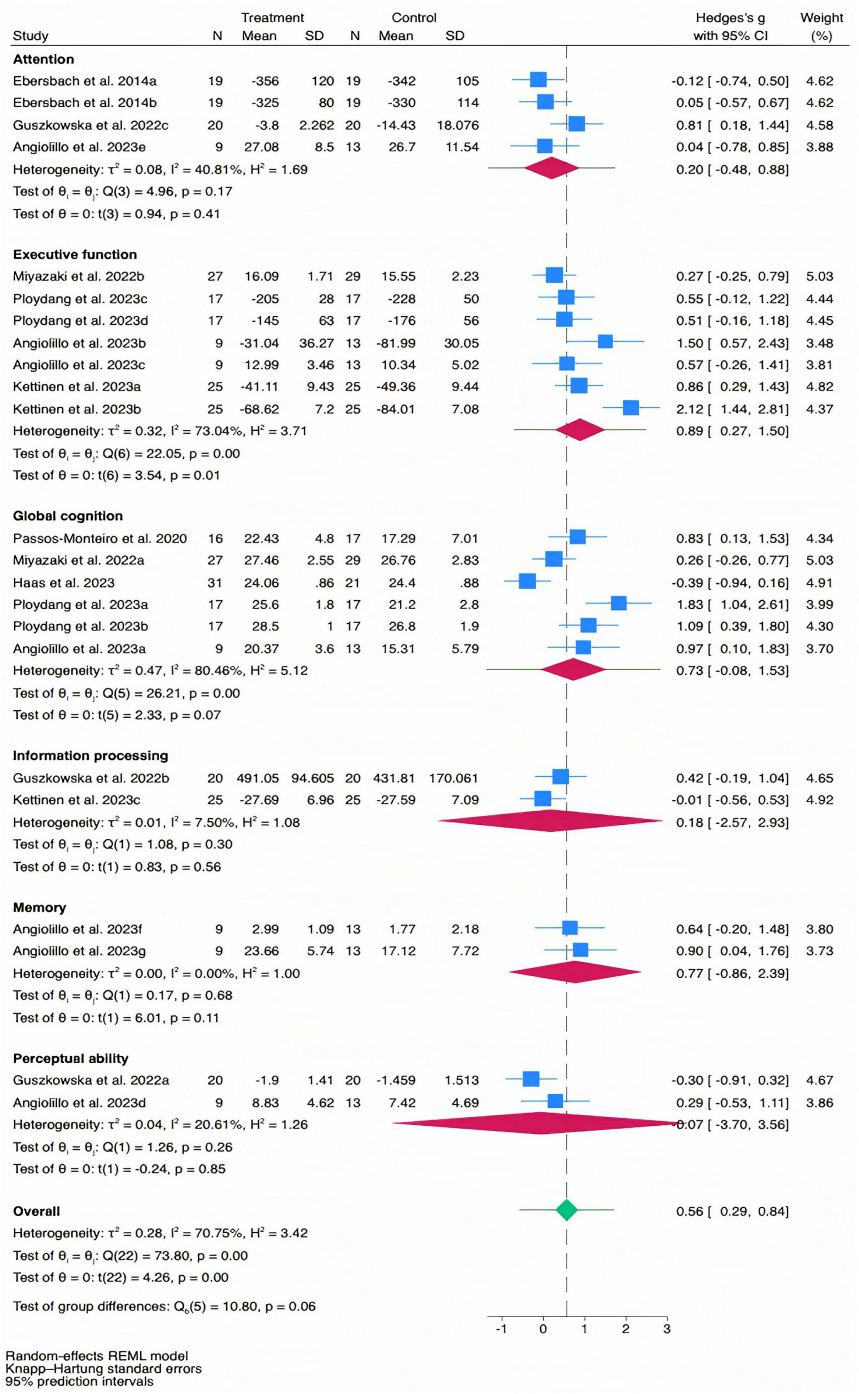
The pooled effect size of NW on cognitive function levels.

**FIGURE 3:**
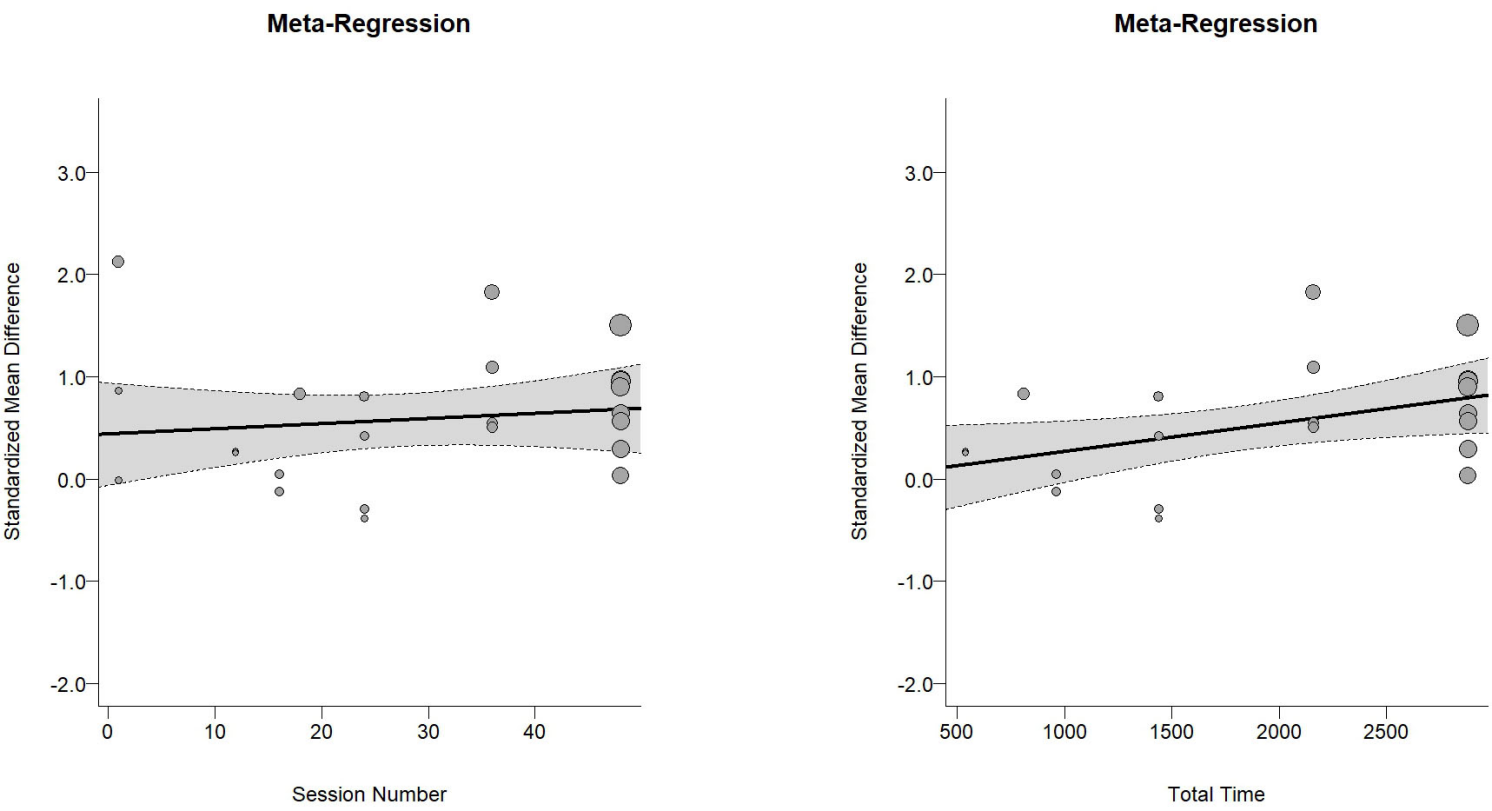
Dose-response curve. **(A)** Session number. **(B)** Total time.

The overall effect size for cognitive function was moderate and statistically significant [Hedges’ *g* = 0.56, 95% CI (0.29, 0.84), PI (−0.57, 1.70), *p* < 0.01, Figure 2], with substantial heterogeneity (I^2^ = 70.75%, *p* < 0.01). Meta-regression analysis indicated no significant association between effect size and the number of intervention sessions (*b* = 0.042, *p* = 0.62, Figure 3A), while it revealed a significant positive association between effect size and the total intervention duration (*b* = 0.0003, *p* = 0.049, Figure 3B).

**FIGURE 4:**
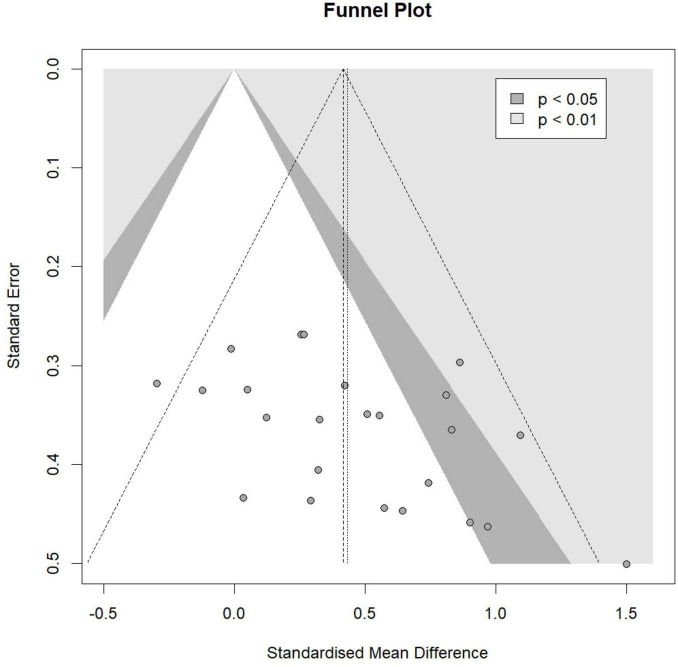
Funnel plot.

For specific cognitive abilities, the effect size for executive function was large and significant [Hedges’ *g* = 0.89, 95% CI (0.27, 1.50), *p* = 0.01], with substantial heterogeneity (I^2^ = 73.04%, *p* < 0.01). The effect size for memory function was moderate but not statistically significant [Hedges’ *g* = 0.77, 95% CI (−0.86, 2.39), p = 0.11], with non-significant heterogeneity (I^2^ = 0.00%, *p* = 0.68). The effect size for global cognition was moderate but not statistically significant [Hedges’ *g* = 0.73, 95% CI (−0.08, 1.53), *p* = 0.07], with substantial heterogeneity (I^2^ = 80.46%, *p* < 0.01). The effect size for attention was small and not statistically significant [Hedges’ *g* = 0.20, 95% CI (−0.48, 0.88), *p* = 0.41], with moderate heterogeneity (I^2^ = 40.81%, *p* = 0.17). The effect size for information processing was trivial and not statistically significant [Hedges’ *g* = 0.18, 95% CI (−2.57, 2.93), *p* = 0.56], with non-significant heterogeneity (I^2^ = 7.50%, *p* = 0.30). The effect size for perceptual ability was trivial and not statistically significant [Hedges’ *g* = −0.07, 95% CI (−3.70, 3.56), *p* = 0.85], with non-significant heterogeneity (I^2^ = 20.61%, *p* = 0.26). The funnel plot (Figure 4) and Egger’s test (*t* = 2.47, *p* = 0.02) indicated a potential risk of publication bias, but the Trim and Fill method for sensitive analysis showed that the pooled effect size (Hedges’ *g* = 0.56, *p* < 0.01) was robust.

To examine characteristics that may contribute to the impact of Nordic walking on cognitive function, we conducted a subgroup analysis using a random effects model on studies that reported large effects involving cognitive function (Table 2). The analysis was based on the following study characteristics: participants’ health status (i.e., healthy population vs. diseased population) and control type (i.e., active vs. inactive).

**TABLE 2:** Subgroup analysis results regarding the effects of executive function.

Variables	No. of studies	Hedges’ g (95% CI)	*P*-value	Test of heterogeneity	
				I^2^ (%)	*P*-value
**The health status of participants**					
Diseased	2	0.70 (0.04, 1.35)	0.04^a^	0	0.33
Healthy	2	1.06 (−1.28, 3.40)	0.19	89.73	<0.01
**Control types**					
Active	2	1.06 (−1.28, 3.40)	0.19	89.73	<0.01
Inactive	2	0.70 (0.04, 1.35)	0.04^a^	0	0.33

*^a^*NW group showed a statistically significant improvement (*P* < 0.05).

The subgroup analysis based on participants’ health status revealed a moderate and statistically significant effect size for the disease group [Hedges’ *g* = 0.70, 95% CI (0.04, 1.35), *p* = 0.04], while the healthy group showed a large but non-significant effect size [Hedges’ *g* = 1.06, 95% CI (−1.28, 3.40), *p* = 0.19]. The analysis by control type indicated that NW had a large but non-significant effect size compared to the active intervention control group [Hedges’ *g* = 1.06, 95% CI (−1.28, 3.40), *p* = 0.19]. In contrast, NW showed a moderate and statistically significant effect size when compared to the inactive control group [Hedges’ *g* = 0.56, 95% CI (0.20, 0.92), *p* < 0.01].

### Sensitivity analysis

Of the eight included studies, one study was with a randomized crossover controlled design. To evaluate the impact of this study on the results, we performed a sensitivity analysis by excluding it. The overall effect size remained significant after excluding it [Hedges’ *g* = 0.49, 95% CI (0.23, 0.75), *p* < 0.01], and was consistent with the previous overall effect size [Hedges’ *g* = 0.56, 95% CI (0.29, 0.84), *p* < 0.01]. It was noted that the heterogeneity between studies assessing executive function decreased significantly after excluding this study (before exclusion: I^2^ = 73.04%, *p* ≤ 0.01; after exclusion: I^2^ = 8.42%, *p* = 0.27), suggesting the difference in the duration of the intervention contributed to the inter study heterogeneity. However, the effect size for executive function also remained significant [Hedges’ *g* = 0.56, 95% CI (0.06, 1.06), *p* = 0.04]. These results confirm the robustness of our findings. Furthermore, by eliminating one effect size at a time, we explore the robustness of the pooled results across different cognitive classifications. The sensitivity analysis showed that the results in terms of attention, executive function, information processing, memory and perception were relatively robust (Supplementary Figure 1). In terms of global cognition alone, after excluding a study where the control group engaged in deep-water exercise, the effect size of the meta-analysis showed a significant change. Before exclusion [Hedges’ *g* = 0.73, 95% CI (−0.08, 1.53), *p* = 0.07], after exclusion [Hedges’ *g* = 0.94, 95% CI (0.23, 1.66), *p* = 0.02]. The above results indicate that the differences in the control conditions might be the reason for the relatively sensitive global cognitive effect. Therefore, these results should be treated with caution.

### Risk of bias and GRADE

The methodological assessment details of each included study are presented in Table 3. The overall quality rating of all included studies is high. The overall mean score is 7.13 ± 1.25; high-quality studies account for 100% (8/8). The level of certainty of the evidence was downgraded by one level due to limitations in study bias (Supplementary Table 2).

**TABLE 3:** Quality assessment of included studies (*n* = 8).

References	Eligibility criteria	Random allocation	Concealed allocation	Similar baseline	Participant blinding	Investi- gator blinding	Assessor blinding	Complete- ness of follow-up	Intention to treat	Between group comparisons	Point measures and variability	Total score	Overall quality
Ebersbach et al., 2014	1	1	0	1	0	0	1	1	0	1	1	7	High
Passos-Monteiro et al., 2020	1	1	1	1	0	0	1	1	1	1	1	9	High
Guszkowska et al., 2022	1	1	0	1	0	0	0	0	0	1	1	5	High
Miyazaki et al., 2022	1	1	0	1	0	0	1	1	1	1	1	8	High
Angiolillo et al., 2023	1	1	0	1	0	0	1	0	0	1	1	6	High
Kettinen et al., 2023	1	1	0	1	0	0	0	1	1	1	1	7	High
Ploydang et al., 2023	1	1	0	1	0	0	0	1	1	1	1	7	High
Haas et al., 2024	1	1	0	1	0	0	1	1	1	1	1	8	High

## Discussion

To the best of our knowledge, this study represents the first systematic review and meta-analysis to comprehensively evaluate the effects of NW on cognitive function in older adults. The findings highlight the potential of NW as a non-pharmacological intervention to mitigate age-related cognitive decline with moderate quality of evidence. Eight studies were included, and the overall score was assessed as “high quality”, indicating no risk of bias. The primary results suggest that NW significantly enhances the executive function of older adults but does not substantially improve global function, memory function, attention, information processing, and perceptual ability. Meta-regression analysis revealed a positive correlation between the total duration of the intervention and the cognitive benefits. Subgroup analysis indicated that the effect of NW might be more pronounced in the diseased populations as compared to essential healthy individuals. The knowledge from this work suggests that NW should be carefully considered in future studies for the rehabilitation plans of older adults with health conditions.

NW showed no significant effects on cognitive domains except executive function, consistent with prior studies of standard walking (Chen et al., 2020a; Passos-Monteiro et al., 2020; Tomoto et al., 2021; Lin et al., 2023). The limited cognitive benefits from walking interventions may largely reflect insufficient aerobic intensity, a well-established determinant of exercise-induced neuroplasticity (Mavros et al., 2017; Chen et al., 2020b; Thomas et al., 2020). Although NW increases energy expenditure compared to conventional walking, its intensity probably remains below the threshold required for broad cognitive adaptations, particularly in hippocampal-dependent memory or temporoparietal-mediated perceptual processing (Passos-Monteiro et al., 2020; Nemoto et al., 2021; Angiolillo et al., 2023; Kettinen et al., 2023; Baker et al., 2025). The neurocognitive value of NW appears to stem primarily from its motor complexity rather than absolute intensity. Unlike standard walking, NW requires synchronized upper and lower limb activation through pole propulsion, recruiting supplementary motor areas, premotor cortices, and frontoparietal networks (Schiffer et al., 2006; Erickson et al., 2013; Niemann et al., 2014). These regions are integral to planning, decision-making, and inhibitory control (Liu-Ambrose et al., 2012; Erickson et al., 2013; Voelcker-Rehage and Niemann, 2013; Niemann et al., 2014; Müller et al., 2017). Such enhanced cortical activation may improve synaptic density and white matter integrity, supported by elevated BDNF levels in NW interventions (Gmiat et al., 2018). Additionally, NW’s rhythmic bilateral coordination may stimulate cerebellar-thalamocortical circuits critical for executive function, optimizing neural efficiency for task-switching and error monitoring (Passos-Monteiro et al., 2020). Consequently, NW acts as a dual-task modality that enhances prefrontal efficiency through mechanisms distinct from pure aerobic stimulation. Our meta-regression indicated longer interventions were associated with greater cognitive benefits, suggesting executive improvements emerge relatively quickly with NW, while other domains may require extended exposure to achieve intensity thresholds through cumulative neurotrophic effects (Jasim et al., 2024; Baker et al., 2025).

Subgroup analyses indicated greater cognitive benefits from NW in diseased populations (e.g., Alzheimer’s patients) compared to essential healthy older adults. This difference may reflect compensatory neuroplasticity in individuals with health conditions, where baseline cognitive impairment increases sensitivity to exercise-induced neurotrophic and vascular changes (Kirk-Sanchez and McGough, 2013; Ploydang et al., 2023; Kadiyala et al., 2024; Popescu et al., 2024). For example, the capacity of NW to improve cerebral perfusion through upper limb engagement may counter hypoperfusion in Alzheimer’s pathology, particularly benefiting clinical groups (Sanders et al., 2020; Angiolillo et al., 2023). Healthy older adults may require higher-intensity or cognitively enhanced NW protocols to exceed their neurocognitive reserve thresholds (Zhang et al., 2023). Additionally, NW demonstrated comparable efficacy to active controls (e.g., standard walking) but outperformed inactive controls. This suggests that while the biomechanical advantages of NW improve adherence and physical outcomes like reduced perceived exertion (Angiolillo et al., 2023; Haas et al., 2024), its cognitive benefits may overlap with other exercise modalities. Nevertheless, NW’s scalability, low injury risk, and dual-task potential make it a practical option for older adults with mobility limitations. However, all studies using active controls involved essential healthy participants, making it unclear whether outcomes were influenced primarily by health status or control group type. Further research is required to clarify the relevant conclusions.

### Limitations

The results of this work still need to be taken with caution. First, the number of included studies was relatively limited (*n* = 8), which may potentially affect the statistical power of our meta-analysis and meta-regression results. Additionally, due to the limited number of studies, subgroup analyses to characterize the influences of protocol settings of NW (e.g., appropriate number of sessions, the frequency of intervention, and Intervention intensity) cannot be completed. Furthermore, interpretations regarding underlying mechanisms remain to be validated in future studies due to the small sample size and the limited number of existing studies. Lastly, although sensitivity analyses showed robust results, heterogeneity among the studies may have a potential influence on the interpretation of outcome measures. Nevertheless, the knowledge obtained from this work will help inform the appropriate design of intervention protocols of NW.

## Conclusion

This study suggests that NW has promise to enhance the executive function in older adults with health conditions. Future RCTs with rigorous designs are needed to help obtain more definitive conclusions on the effects of NW on cognitive function in older adults.

## Data Availability

The original contributions presented in this study are included in this article/Supplementary material, further inquiries can be directed to the corresponding authors.
