# Supplementary material for: The effects of Nordic walking on cognitive function in older adults: a systematic review and meta-analysis
Source: Front Aging Neurosci. 2025 Sep 17;17:1666449. doi: 10.3389/fnagi.2025.1666449 (PMC12484218; doi:10.3389/fnagi.2025.1666449)
Supplement: Supplementary file 1 [file Table_1.docx]

Supplementary Material

Table S1: Search form and corresponding results

Table S2: Quality assessment of included studies (n = 8)

Table S3: Cognitive classification and test names

Figure S1: Effect size plot based on the “leave-one-out” method

**Table S1:** Search from and corresponding results

| **Data source** | **Search query** | **Results** |
| --- | --- | --- |
| **Web of science** | ((TS=(Nordic walking OR Nordic pole walking OR pole walking)) AND TS=(Cognitive function OR cognition OR Cognitive performance)) NOT (TS=(Review OR Overview OR Meta)) | 84 |
| **Pubmed** | (Nordic walking OR Nordic pole walking OR pole walking) AND (Cognitive function OR cognition OR Cognitive performance) NOT (Review OR Overview OR Meta) | 64 |
| **Medline** | TX ( Nordic walking OR Nordic pole walking OR pole walking ) AND TX ( Cognitive function OR cognition OR Cognitive performance ) NOT TX ( Review OR Overview OR Meta ) | 29 |
| **SPORT-Discus** | TX ( Nordic walking OR Nordic pole walking OR pole walking ) AND TX ( Cognitive function OR cognition OR Cognitive performance ) NOT TX ( Review OR Overview OR Meta ) | 47 |
| **Cochrane Library** | Title Abstract keyword:("Nordic walking" OR "Nordic pole walking" OR "pole walking") AND ("Cognitive function" OR "cognition" OR "Cognitive performance") NOT ("Review" OR "Overview" OR "Meta-analysis") | 46 |
| **PsycINFO** | TX ( Nordic walking OR Nordic pole walking OR pole walking ) AND TX ( Cognitive function OR cognition OR Cognitive performance ) NOT TX ( Review OR Over-view OR Meta ) | 3 |
| **Scopus** | ( ALL ( Nordic walking OR Nordic pole walking OR pole walking ) AND ALL ( Cognitive function OR cognition OR Cognitive performance ) AND NOT TITLE-ABS-KEY ( Review OR Overview OR Meta ) ) AND ( LIMIT-TO ( DOCTYPE , "ar" ) ) | 61 |

**Table S2:** Quality assessment of included studies (n = 8)

| Quality assessment | | | | | | | No of participants | | Effect | Quality | Importance |
| --- | --- | --- | --- | --- | --- | --- | --- | --- | --- | --- | --- |
| No of experiments | **Design** | **Risk of bias** | **Inconsistency** | **Indirectness** | **Imprecision** | **Other considerations** | **NW** | **Control** | **Absolute** |  |  |
| 8 | randomised trials | serious^a^ | no serious inconsistency | no serious indirectness | no serious imprecision | none | 166 | 161 | Hedges’g 0.56 higher (0.29 higher to 0.84 higher) | ⊕⊕⊕⊝ | CRITICAL |
|  |  |  |  |  |  |  |  |  |  | MODERATE |  |

^a^ Risk of blinding

**Table S3:** Cognitive classification and test names

| **Cognitive ability category** | **Test name** |
| --- | --- |
| Global cognitive function | MoCA (Montreal Cognitive Assessment) MMSE (Mini Mental State Examination) |
| Memory function | RVLT-D (Rey’s auditory Verbal Learning Test-Delayed Recall) RVLT-I (Rey’s auditory Verbal Learning Test-Immediate Recall) |
| Executive function | FAB (Frontal Assessment Battery) SWCT (Stroop Word-Color Interference test) SCT (Stroop Color test) TMT-B (Trail Making Test part B) TMT B-A (Trail Making Test part B-A) |
| Perceptual abilities | CGD (Copying Geometric Drawings) APT 3/8 (Attention and Perceptivity Test, version 3/8, perception fallibility) |
| Information processing speed | TMT-A (Trail Making Test part A) APT 3/8 (Attention and Perceptivity Test, version 3/8, perception speed) |
| Attention | Attentional Matrices nRT (Cued reaction time) cRT (noncued reaction time) APT 3/8 (Attention and Perceptivity Test, version 3/8, attention fallibility) |


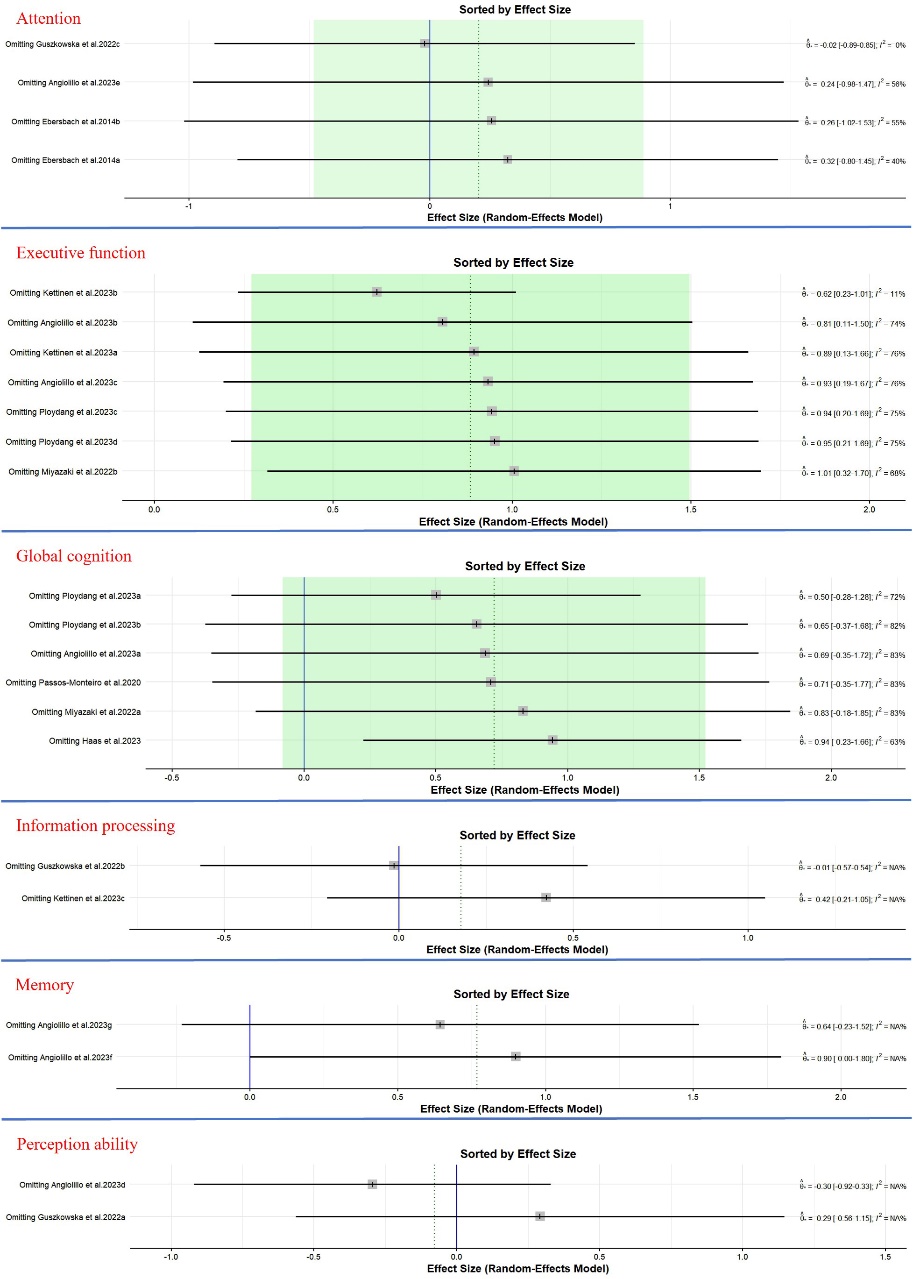


**Figure S1**: Effect size plot based on the "leave-one-out" method
